# ﻿Three new species of *Favolaschia* (Mycenaceae, Agaricales) from South China

**DOI:** 10.3897/mycokeys.104.117310

**Published:** 2024-04-16

**Authors:** Qiu-Yue Zhang, Hong-Gao Liu, Wan-Ying Li, Xin Zhang, Yu-Cheng Dai, Fang Wu, Lu-Sen Bian

**Affiliations:** 1 State Key Laboratory of Efficient Production of Forest Resources, School of Ecology and Nature Conservation, Beijing Forestry University, Beijing 100083, China Beijing Forestry University Beijing China; 2 Yunnan Key Laboratory of Gastrodia and Fungi Symbiotic Biology, Zhaotong University, Zhaotong 657000, Yunnan, China Zhaotong University Yunnan China; 3 Experimental Centre of Forestry in North China, Warm Temperate Zone Forestry Jiulong Mountain National Permanent Scientific Research Base, Chinese Academy of Forestry, Beijing 102300, China Chinese Academy of Forestry Beijing China

**Keywords:** New taxa, phylogeny, taxonomy, wood-decaying fungi

## Abstract

The genus *Favolaschia* within the family Mycenaceae is characterised by the gelatinous basidiomata with poroid hymenophore and most species inhabit monocotyledonous plants. In this study, many samples covering a wide geographic range in China were examined morphologically and phylogenetically using concatenated ITS1-5.8S-ITS2-nLSU sequence data. Three new species clustering in Favolaschiasect.Anechinus, namely *Favolaschiaimbricata*, *F.miscanthi* and *F.sinarundinariae*, are described. *Favolaschiaimbricata* is characterised by imbricate basidiomata with pale grey to greyish colour when fresh and broadly ellipsoid basidiospores measuring 7–9 × 5–6.8 µm; *F.miscanthi* is characterised by satin white basidiomata when fresh, broadly ellipsoid basidiospores measuring 7.5–10 × 5.5–7 µm and inhabit rotten *Miscanthus*; *F.sinarundinariae* is characterised by greyish-white basidiomata when fresh, dark grey near the base upon drying, broadly ellipsoid to subglobose basidiospores measuring 7–9 × 5–7 µm and inhabit dead *Sinarundinaria*. The differences amongst the new species and their morphologically similar and phylogenetically related species are discussed. In addition, an updated key to 19 species of *Favolaschia* found in China is provided.

## ﻿Introduction

The genus *Favolaschia* (Pat.) Pat., typified by *F.gaillardia* (Pat.) Pat. (Patouillard 1887), belongs to the Mycenaceae Overeem (Agaricales) (Moncalvo et al. 2002; Bodensteiner et al. 2004). It is characterised by gelatinous basidiomata with poroid hymenophore, a monomitic hyphal system, a gelatinous hyphal structure, the presence of gloeocystidia and acanthocystida which are terminal, mostly swollen tips of hyphae covered by outgrowths (Clémençon 1977) and amyloid basidiospores (Singer 1945, 1974; Gillen et al. 2012; Capelari et al. 2013; Magnago et al. 2013; Zhang and Dai 2021; Zhang et al. 2023). *Favolaschia* species have a worldwide distribution and prefer humid climates with the highest species diversity in subtropical to tropical zones (Singer 1974; Gillen et al. 2012; Magnago et al. 2013). The basidiomata are found mostly on decaying parts of monocotyledonous plants, as well as ferns and cause white rot (Singer 1974; Gillen et al. 2012).

A total of 119 records of *Favolaschia* are listed in Index Fungorum (http://www.indexfungorum.org) and around 60 species are accepted (Johnston et al. 2006; Gillen et al. 2012; Magnago et al. 2013). The name “*Favolaschia*” was first introduced by Patouillard (1887) as a section of *Laschia* Fr and treated at a generic level later (Patouillard and Lagerheim 1892). The taxonomic history of the genus has been systematically compiled by Singer (1974) and Johnston et al. (2006). Singer (1974) divided *Favolaschia* into two sections, based on morphological studies, namely section Favolaschia Singer and section Anechinus Singer. Amongst them, taxa of F.sect.Favolaschia is characterised by having mostly orange, yellow, red, lateritious or pink pileus and the presence of acanthocytes in pileipellis. Inversely, taxa of F.sect.Anechinus are characterised by lacking acanthocystidia or replaced by diverticulate hyphae. Later, Johnston et al. (2006), Gillen et al. (2012) and Magnago et al. (2013) confirmed the two sections by phylogenetic analyses.

Recently, the systematic study of *Favolaschia* in China has gradually increased. Tolgor et al. (2021) investigated the phylogenetic relationships of *Favolaschia* and its related genera in Mycenaceae and reported four species distributed in China. Zhang and Dai (2021) investigated phylogenetic analysis of the genus *Favolaschia*, based on a large sample and revealed four new species in the *F.calocera* complex. Until Zhang et al. (2023) re-summarised the species diversity, phylogenetic relationships, divergence time and potential geographic distribution using a large number of samples covering a wide geographic range in China. To date, 16 species of *Favolaschia* have been reported from China, based on phylogenetic analyses, namely, *F.brevibasidiata* Q.Y. Zhang and Y.C. Dai, *F.brevistipitata* Q.Y. Zhang and Y.C. Dai, *F.longistipitata* Q.Y. Zhang and Y.C. Dai, *F.minutissima* Q.Y. Zhang and Y.C. Dai, *F.bannaensis* Q.Y. Zhang & Y.C. Dai, *F.crassipora* Q.Y. Zhang & Y.C. Dai, *F.flabelliformis* Q.Y. Zhang & Y.C. Dai, *F.manipularis* (Berk.) Teng, *F.peziziformis* (Berk. and M.A. Curtis) Kuntze, *F.pustulosa* (Jungh.) Kuntze, *F.rigida* Q.Y. Zhang & Y.C. Dai, *F.semicircularis* Q.Y. Zhang & Y.C. Dai, *F.subpustulosa* Q.Y. Zhang & Y.C. Dai, *F.tenuissima* Q.Y. Zhang & Y.C. Dai, *F.tephroleuca* Q.Y. Zhang & Y.C. Dai and *F.tonkinensis* (Pat.) Kuntze; the first four species belong to the *F.calocera* complex of the F.sect.Favolaschia; while the others belong to the F.sect.Anechinus (Zhang and Dai 2021; Guo et al. 2022; Ma et al. 2022; Wu et al. 2022a; Dong et al. 2023; Zhang et al. 2023).

During a study on wood-rotting fungi from south China, several samples belonging to *Favolaschia* were collected and three unknown species were morphologically distinguished. To confirm the affinity of the taxa, phylogenetic analysis was performed, based on a combined sequence dataset of ITS1-5.8S-ITS2-nLSU.

## ﻿Materials and methods

### ﻿Morphological studies

**Figure 1.**
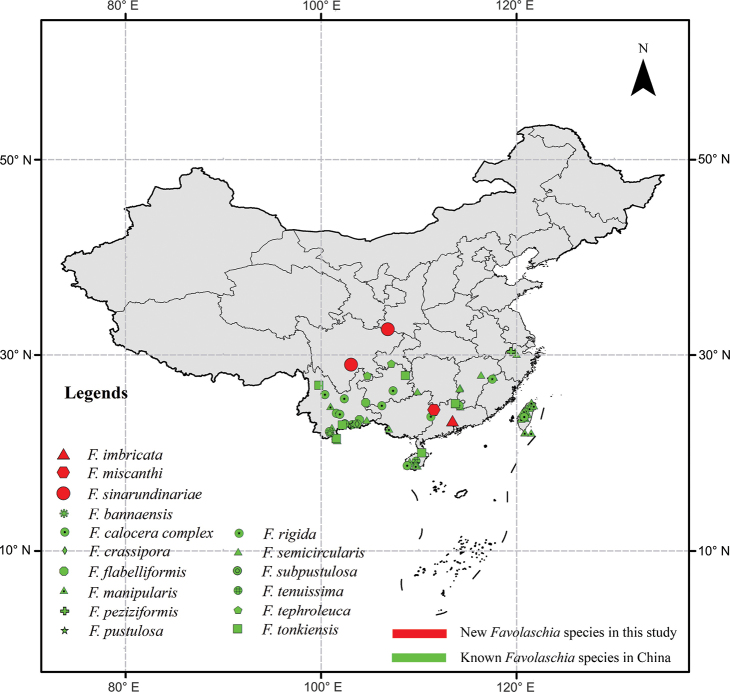
The geographical locations of *Favolaschia* species distributed in China (the base map of China was obtained from the Map Technology Review Center, Department of Natural Resources, http://bzdt.ch.mnr.gov.cn/).

The specimens were collected from Guangxi Autonomous Region, Guangdong and Sichuan Provinces in south China and the specific locations were marked in Fig. 1. They were deposited in the Fungarium of the Institute of Microbiology, Beijing Forestry University (BJFC), Beijing, China. Macro-morphological descriptions were based on field notes and dried specimens. Microscopic features were examined and described in 5% KOH (potassium hydroxide) and 2% phloxine B (C_20_H_2_Br_4_Cl_4_Na_2_O_5_) with a magnification of up to 1,000× using a Nikon Eclipse 80i microscope and phase contrast illumination. Colour terms followed Kornerup and Wanscher (1978) and Petersen (1996). A Nikon Digital Sight DS-L3 camera was used to photograph microscopic structures. Other reagents, including Cotton Blue and Melzer’s reagents were used to observe the micromorphology structures following Zhang et al. (2023). To show the variation in spore sizes, 5% of measurements were excluded from each end of the range and shown in parentheses. Thirty basidiospores from each specimen were measured. The following abbreviations are used: IKI = Melzer’s reagent, IKI+ = amyloid; CB = Cotton Blue, CB– = acyanophilous in Cotton Blue; L = arithmetic average of basidiospores length, W = arithmetic average of basidiospores width, Q = L/W ratios, (*n* = x/y) = the number of spores (x) measured from a given number of specimens (y).

### ﻿DNA extraction and sequencing

A cetyl trimethylammonium bromide (CTAB) rapid plant genome extraction kit (Aidlab Biotechnologies, Co., Ltd., Beijing, China) was used to extract DNA (Wu et al. 2020, 2022b). The ITS1-5.8S-ITS2 region was amplified with the primer pair ITS5/ITS4 (White et al. 1990) using the following protocol: initial denaturation at 95 °C for 4 min, followed by 34 cycles at 94 °C for 40 s, 54 °C for 45 s and 72 °C for 1 min and final extension at 72 °C for 10 min. The nLSU region was amplified with the primer pair LR0R/LR7 (White et al. 1990) using the following protocol: initial denaturation at 94 °C for 1 min, followed by 34 cycles at 94 °C for 30 s, 50 °C for 1 min and 72 °C for 1.5 min and final extension at 72 °C for 10 min. The PCR products were purified and sequenced by the Beijing Genomics Institute (BGI), China with the same primers. The newly-generated sequences in this study have been deposited in GenBank and are listed in Table 1.

**Table 1.** Names, specimen numbers, locality and corresponding GenBank accession numbers of the taxa used in this study.

Species	Specimen no.	Locality	ITS no.	nLSU no.
*Favolaschiaandina*	KG0025	Panama	HM246678	HM246679
*F.aurantiaca*	FK2047	Brazil	JX987670	–
*F.aurantiaca*	KG0013	Panama	–	HM246676
*F.auriscalpium*	Isolate 5	–	KY649461	–
*F.auriscalpium*	TH1018	Guyana	DQ026241	–
*F.austrocyatheae*	PDD75609	New Zealand	NR132809	–
*F.austrocyatheae*	PDD75609	New Zealand	DQ026257	–
*F.bannaensis*	Dai 22587	Yunnan, China	ON870497	ON870473
*F.bannaensis*	Dai 22589	Yunnan, China	ON870498	ON870474
*F.bannaensis*	Dai 22590	Yunnan, China	ON870499	ON870475
*F.brevibasidiata*	Cui 6573	Hainan, China	MZ661794	–
*F.brevibasidiata*	JM98186	Yunnan, China	DQ026239	–
*F.brevistipitata*	Dai 19780	Yunnan, China	MZ661772	MZ661742
*F.brevistipitata*	Dai 19855	Yunnan, China	MZ661773	MZ661743
*F.brevistipitata*	Dai 19856	Yunnan, China	MZ661774	MZ661744
*F.calocera*	PC99060	Madagascar	DQ26252	–
*F.calocera*	PC99497	Madagascar	DQ026253	–
*F.cinnabarina*	Isolate 4421	Brazil	JX987669	–
*F.cinnabarina*	RVPR82	–	–	AF261416
*F.claudopus*	Dai 18656	Australia	MZ661775	MZ661735
*F.claudopus*	Dai 18663	Australia	MZ661776	MZ661734
*F.claudopus*	SR346	Kenya	DQ026237	–
*F.claudopus*	PDD74554	New Zealand	DQ026251	–
*F.claudopus*	PDD75323	New Zealand	DQ026248	–
*F.claudopus*	PDD75686	New Zealand	DQ026249	–
*F.claudopus*	DUKE2952	New Zealand	DQ026238	–
*F.crassipora*	Dai 19769	Yunnan, China	ON870500	ON870476
*F.crassipora*	Dai 19871	Yunnan, China	ON870501	ON870477
*F.cyatheae*	PDD75316	New Zealand	NR132808	–
*F.cyatheae*	PDD75316	New Zealand	DQ026256	–
*F.dealbata*	KG0015	Panama	–	HM246677
*F.flabelliformis*	Dai 20010	Yunnan, China	ON870502	ON870478
*F.flabelliformis*	Dai 20016	Yunnan, China	ON870503	–
*F.flabelliformis*	Dai 24354	Yunnan, China	**OR271272**	**OR260441**
*F.heliconiae*	KG0026	Panama	–	HM246680
***F.imbricata***	**Dai 24702**	**Guangdong, China**	**OR334876**	–
***F.imbricata***	**Dai 24703**	**Guangdong, China**	**OR334877**	**OR334881**
***F.imbricata***	**Dai 24704**	**Guangdong, China**	**OR334878**	**OR334882**
*F.longistipitata*	Dai 13221	Yunnan, China	MZ661777	–
*F.longistipitata*	Dai 13226	Yunnan, China	MZ661778	–
*F.longistipitata*	Cui 11128	Yunnan, China	MZ661779	–
*F.longistipitata*	Dai 17597	Yunnan, China	MZ661780	–
*F.longistipitata*	Dai 17598	Yunnan, China	MZ661781	–
*F.longistipitata*	Dai 17601	Yunnan, China	MZ661782	–
*F.longistipitata*	Dai 19799	Yunnan, China	MZ661784	MZ661739
*F.longistipitata*	Dai 19893	Yunnan, China	MZ661785	MZ661740
*F.longistipitata*	Dai 20019	Yunnan, China	MZ661786	MZ661741
*F.longistipitata*	Dai 20328	Yunnan, China	MZ661787	–
*F.longistipitata*	Dai 20341	Yunnan, China	MZ661788	–
*F.longistipitata*	Dai 20355	Yunnan, China	MZ661789	–
*F.luteoaurantiaca*	Isolate 4475	–	JX987667	–
*F.luteoaurantiaca*	SP445750	Brazil	NR132874	–
*F.macropora*	KG0027	Panama	NR132845	HM246682
*F.manipularis*	Dai 20612	Yunnan, China	MZ801776	MZ914395
*F.manipularis*	Dai 20653	Yunnan, China	MZ801777	–
*F.minutissima*	JM98372	Thailand	DQ026240	–
*F.minutissima*	Dai 10753	Hainan, China	MZ661790	–
*F.minutissima*	Dai 20085	Hainan, China	MZ661791	MZ661736
*F.minutissima*	Dai 20086	Hainan, China	MZ661792	MZ661737
*F.minutissima*	Dai 20088	Hainan, China	MZ661793	MZ661738
***F.miscanthi***	**Dai 24652**	**Guangxi, China**	**OR334879**	**OR334883**
***F.miscanthi***	**Dai 24653**	**Guangxi, China**	**OR334880**	**OR334884**
*F.peziziformis*	ICMP1575	Japan	DQ026255	–
*F.peziziformis*	PDD67440	New Zealand	–	AY572008
*F.pustulosa*	PDD75686	New Zealand	DQ026254	–
*F.pustulosa*	Dai 19758	Yunnan, China	MT292325	MT293226
*F.rigida*	Dai 18566A	Guangxi, China	ON870504	ON870479
*F.rigida*	Dai 20764	Yunnan, China	ON870505	ON870480
*F.rigida*	Dai 22614	Fujian, China	ON870506	–
*F.semicircularis*	Dai 19725	Guangdong, China	ON870507	ON870481
*F.semicircularis*	Dai 19923	Yunnan, China	ON870508	ON870482
*F.semicircularis*	Dai 19936	Yunnan, China	ON870509	ON870483
*F.semicircularis*	Dai 19939	Yunnan, China	ON870510	–
*F.semicircularis*	Dai 19980	Yunnan, China	ON870511	ON870484
*F.semicircularis*	Dai 19981	Yunnan, China	ON870512	ON870485
*F.semicircularis*	Dai 22290	Zhejiang, China	ON870513	ON870486
*F.semicircularis*	Dai 22298	Zhejiang, China	ON870515	ON870488
*F.semicircularis*	Dai 22302	Zhejiang, China	ON870516	–
*F.semicircularis*	Dai 23702	Hunan, China	**OR271273**	**OR260442**
*F.semicircularis*	Dai 24689	Guangxi, China	**OR271274**	**OR260443**
*F.semicircularis*	Dai 24748	Jiangxi, China	**OR271275**	**OR260444**
*F.semicircularis*	Dai 24687	Guangxi, China	**OR271276**	**OR260445**
*F.semicircularis*	Dai 22383	Fujian, China	ON870517	–
***F.sinarundinariae***	**Dai 26115**	**Sichuan, China**	**OR575908**	**OR575906**
***F.sinarundinariae***	**Dai 26116**	**Sichuan, China**	**OR575909**	**OR575907**
***F.sinarundinariae***	**Dai 26123**	**Sichuan, China**	**OR855969**	**OR855975**
***F.sinarundinariae***	**Dai 26129**	**Sichuan, China**	**OR855970**	–
***F.sinarundinariae***	**Dai 26130**	**Sichuan, China**	**OR855971**	–
***F.sinarundinariae***	**Dai 26131**	**Sichuan, China**	**OR855972**	–
***F.sinarundinariae***	**Dai 26134**	**Sichuan, China**	**OR855973**	**OR855976**
***F.sinarundinariae***	**Dai 26140**	**Sichuan, China**	**OR855974**	**OR855975**
*F.* sp. 1	DUKE2708	Australia	DQ026234	–
*F.* sp. 1	DUKE2876	Australia	DQ026235	–
*F.* sp. 1	DUKE3195	Papua New Guinea	DQ026236	–
*F.* sp. 2	Isolate 4550	Panama	JX987668	–
*F.sprucei*	TH6418	Guyana	DQ026246	–
*F.subpustulosa*	Dai 20719	Yunnan, China	ON870518	ON870489
*F.tenuissima*	Dai 22072	Hainan, China	ON870520	ON870491
*F.tenuissima*	Dai 22071	Hainan, China	ON870519	ON870490
*F.tephroleuca*	Dai 22282	Yunnan, China	ON870521	ON870492
*F.tephroleuca*	Dai 22288	Chongqing, China	ON870522	ON870493
*F.tonkinensis*	Dai 21955	Hainan, China	ON870523	ON870494
*F.tonkinensis*	Dai 21956	Hainan, China	ON870524	ON870495
*F.tonkinensis*	Dai 21964	Hainan, China	ON870525	–
*F.tonkinensis*	Dai 21965	Hainan, China	ON870526	ON870496
*F.tonkinensis*	Dai 21966	Hainan, China	ON870527	–
*F.tonkinensis*	Dai 19704	Guangdong, China	**OR271277**	**OR260446**
*F.tonkinensis*	BCC 18686	–	MN093316	MN093317
*F.tonkinensis*	JM98229	Yunnan, China	DQ026247	–
*F.varariotecta*	DUKE3893	Puerto Rico	DQ026243	–
*F.varariotecta*	DUKE4038	Puerto Rico	DQ026244	–
*F.xtbgensis*	HKAS 121667	Yunnan, China	OL413048	OL413044
*F.xtbgensis*	HKAS 121975	Yunnan, China	OL413036	OL413035
*Mycenaseminau*	ACL136	Malaysia	KF537250	KJ206952
*Mycenaseminau*	ACL308	Malaysia	KF537252	KJ206964

Notes: New sequences are in bold; “–” represents missing data.

### ﻿Phylogenetic analyses

The dataset of concatenated ITS1-5.8S-ITS2-nLSU sequences of the *Favolaschia* was analysed. Sequences of *Mycenaseminau* A.L.C. Chew & Desjardin were used as outgroups (Chew et al. 2014). The newly-generated sequences in this study and additional sequences retrieved from Zhang et al. (2023) were partitioned to ITS1, 5.8S, ITS2, nrLSU and then aligned separately using MAFFT v.74 (http://mafft.cbrc.jp/alignment/server/, Katoh et al. 2017) with the G-INS-I iterative refinement algorithm and optimised manually in BioEdit v.7.0.5.3 (Hall 1999). The separate alignments were then concatenated using PhyloSuite v.1.2.2 (Zhang et al. 2020).

Maximum Likelihood (ML) analyses and Bayesian Inference (BI) were carried out by using RAxML v.8.2.10 (Stamatakis 2014) and MrBayes 3.2.6 (Ronquist and Huelsenbeck 2003), respectively. In ML analysis, statistical support values were obtained by using rapid bootstrapping with 1000 replicates, with default settings for other parameters. For BI, the best-fit partitioning scheme and substitution model were determined by using ModelFinder (Kalyaanamoorthy et al. 2017) via the “greedy” algorithm, branch lengths estimated as “linked” and AICc. Four Markov chain Monte Carlo chains (one cold) were constructed for 5,000,000 generations, with sampling every 1000 generations. Convergence was assessed as the standard deviation of split frequencies < 0.01. The first quarter of the trees, which represented the burn-in phase of the analyses, were discarded and the remaining trees were used to calculate posterior probabilities (BPP) in the majority rule consensus tree.

Phylogenetic trees were visualised by using FigTree version 1.4.4 (Rambaut 2018). Branches that received bootstrap supports for ML (≥ 75%) and BPP (≥ 0.95) were considered as significantly supported. The best topologies from ML analyses are shown in this study and the final alignments and the retrieved topologies were deposited in TreeBASE (http://treebase.org/treebase-web/home.html), under accession ID: 30973.

## ﻿Results

### ﻿Phylogenetic analyses

**Figure 2.**
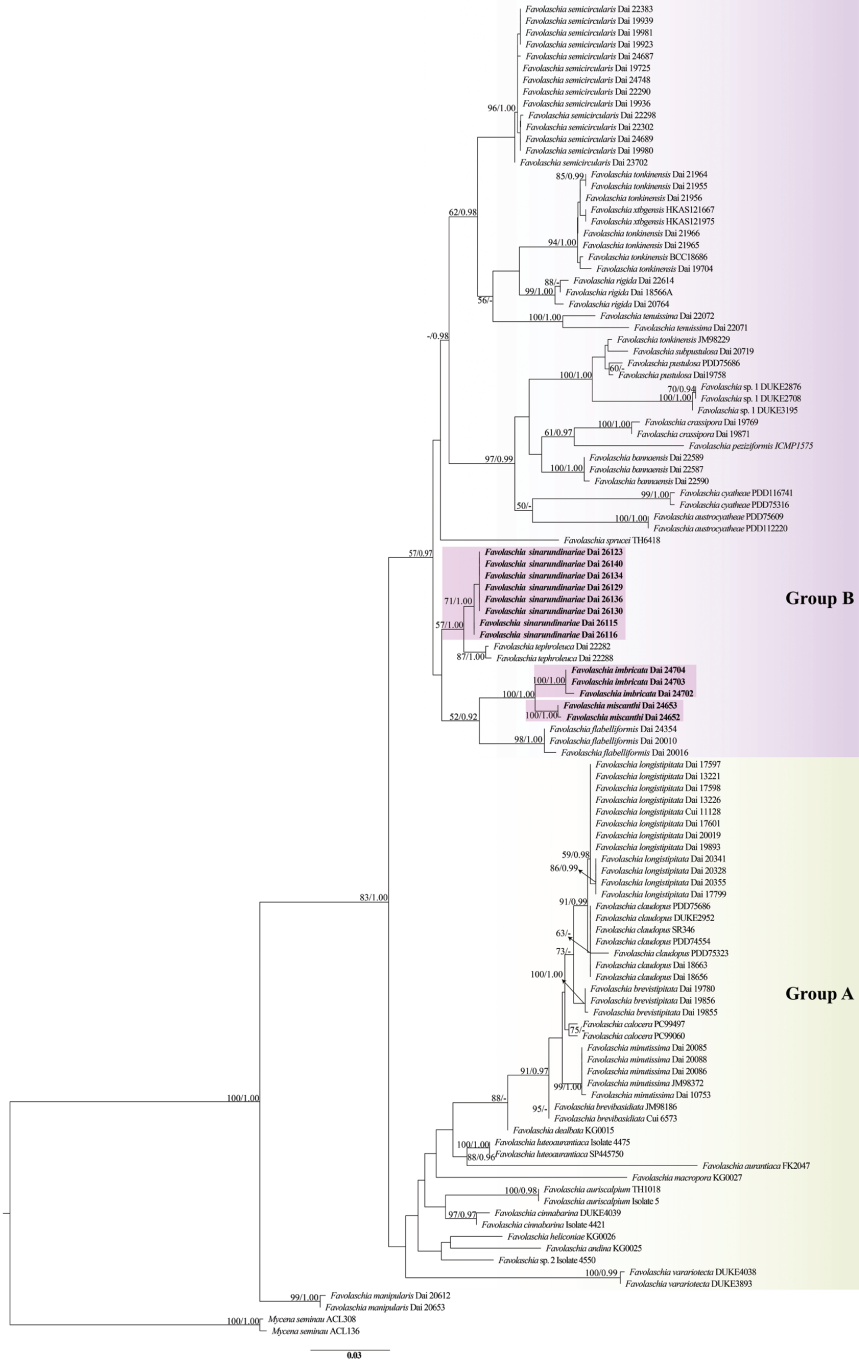
Maximum Likelihood (ML) tree illustrating the phylogeny of *Favolaschia*, based on a combined ITS1-5.8S-ITS2-nLSU-mtSSU-nuSSU dataset. Branches are labelled with parsimony bootstrap values (ML) higher than 50% and Bayesian Posterior Probabilities (BPPs) more than 0.90. (Group A: Favolaschiasect.Favolaschia; Group B: Favolaschiasect.Anechinus)

In this study, the combined ITS1-5.8S-ITS2-nLSU dataset included sequences from 113 specimens, representing 34 species of *Favolaschia* and one species of *Mycena* (Pers.) Roussel as the outgroup (Table 1, Fig. 2). ModelFinder suggested models were HKY+F+G4 for ITS1+ITS2, K2P+G4 for 5.8s and K2P+G4 for nLSU, for the Bayesian analysis. The BI analysis resulted in a concordant topology with an average standard deviation of split frequencies of 0.008584. The ML and BI analyses resulted in nearly identical topologies and only the ML tree is presented with the bootstrap supports for ML and BPP not less than 50% and 0.90, respectively.

The phylogeny is similar to those of Johnston et al. (2006), Magnago et al. (2013) and Zhang et al. (2023). All *Favolaschia* samples are clustered within two groups (A and B), which correspond to the sections proposed by Singer (1974): Favolaschiasect.Favolaschia (group A) and F.sect.Anechinus (group B), except *F.manipularis.* In this study, three new lineages with high support (100/1.00, 100/1.00 and 71/1.00, respectively) nest in F.sect.Anechinus (group B). Amongst them, five specimens from Guangdong and Guangxi formed two lineages and clustered into a clade with strong support (100/1.00), namely *F.imbricata* and *F.miscanthi*. Eight specimens from Sichuan formed a support lineage, namely *F.sinarundinariae*, sister to *Favolaschiatephroleuca*. Furthermore, similarity searches with the ITS barcoding from the three new species were performed using BLAST (http://www.ncbi.nlm.nih.gov/BLAST/) from NCBI and showed less than 95% identity, respectively, in the genus *Favolaschia*, which demonstrated the obvious nucleotide differences of the three species with other species in the genus.

### ﻿Taxonomy

#### ﻿ Favolaschia imbricata

Taxon classificationFungiAgaricalesMycenaceae

Q.Y. Zhang, L.S. Bian, F. Wu & Y.C. Dai sp. nov.

271D0C10-55C2-5D45-86BC-275140DCFED3

851807

Figs 3A , 4

##### Type.

China, Guangdong Province, Guangzhou, Baiyun District, Maofengshan Forest Park, dead bamboo, 15 Apr 2023, Dai 24702 (BJFC042256, holotype).

##### Etymology.

“*imbricata*” (Lat.): refers to the species having imbricate basidio­mata.

##### Macrostructures.

Basidiomata annual, gregarious, gelatinous when fresh and dry. Pilei 2–5 × 1.5–3 mm, conchoid, semicircular to subcircular; pileal surface pale grey to greyish-white (B1) when fresh, dark grey or mouse-grey (1D1–1F1) upon drying, convex with a reticulate pattern matching the pores below, faintly pruinose when dry; margin incurved, entire; context thin. Hymenophore paler than pileal surface, greyish-white (B1) when fresh and ash-grey (1B1–1C1) when dry, poroid, about 22–55 pores per basidiomata; mature pores 0.2–0.6 mm diam., polygonal, larger near the base and smaller near the edge, the marginal pores often incomplete; tubes up to 0.3 mm long. Stipe absent.

**Figure 3.**
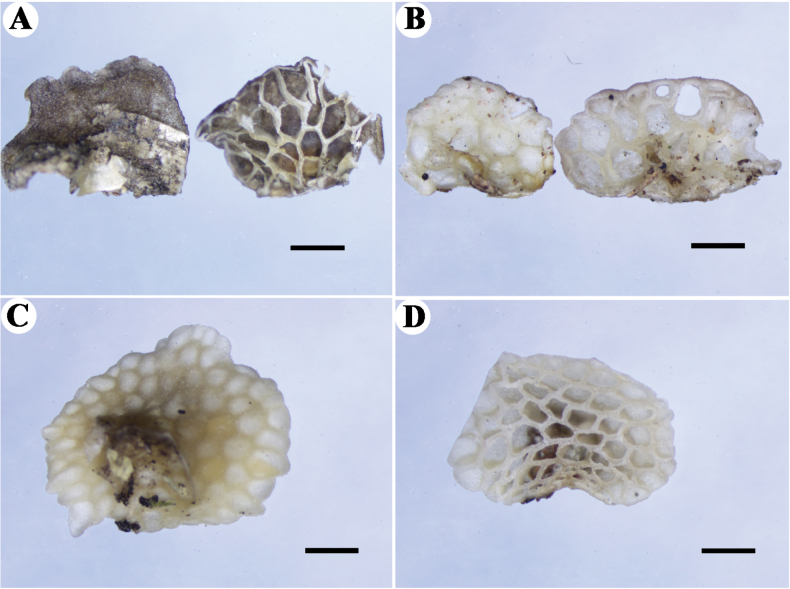
Dry basidiomata of *Favolaschia* species **A***Favolaschiaimbricata***B***Favolaschiamiscanthi***C, D***Favolaschiasinarundinariae.* Scale bars: 1 mm.

##### Microstructures.

Basidiospores (6.8–)7–9 × 5–6.8 µm, L = 7.92 μm, W = 5.84 μm, Q = 1.31–1.38 (*n* = 90/3), broadly ellipsoid to subglobose, hyaline, thin-walled, smooth, with some guttules, faintly IKI+, CB–. Basidia 24–30 × 7–13 μm, cylindrical or clavate with some guttules, 4–spored, sterigmata 3–6 μm long; basidioles similar in shape to the basidia, but slightly smaller. Gloeocystidia and acanthocystidia absent. Cheilocystidia 25–39 × 7–14 μm, present at dissepiment edge, cylindrical or clavate, with obtuse diverticulate projections at the apex, thin-walled. Pileipellis hyphae subparallel, smooth to diverticulate, thin-walled, 5–11 µm diam.; terminal cells cystidioid or pyriform, smooth, thin-walled. Tramal hyphae subparallel, widely spaced in a gelatinous matrix, some with dense contents, some collapsed, thin-walled, 2–5 μm diam. Clamp connections present.

**Figure 4.**
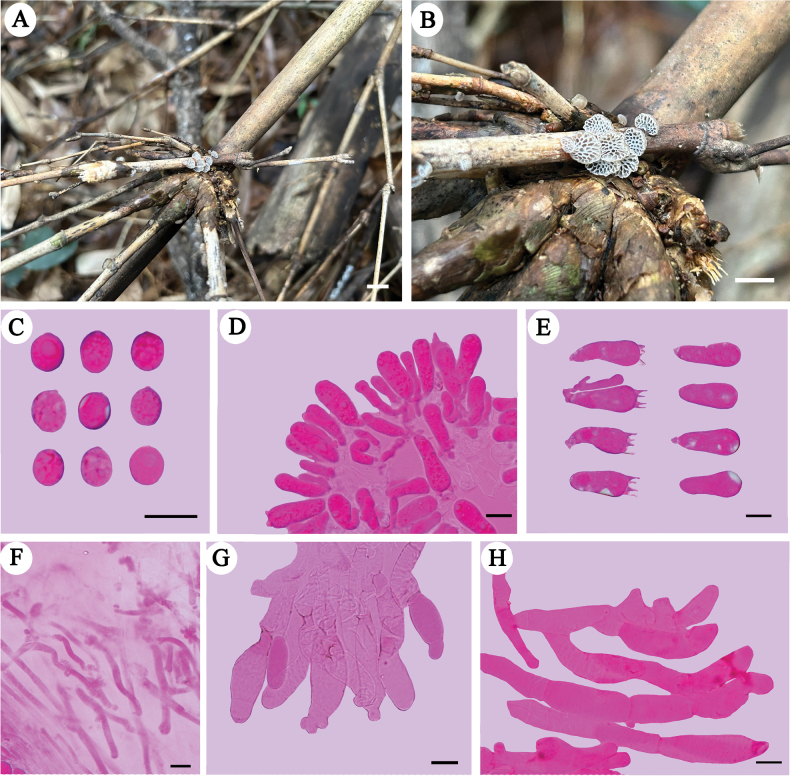
Basidiomata and microscopic structures of *Favolaschiaimbricata***A, B** basidiomata (Dai 24702, holotype) **C** basidiospores **D** hymenium in trama **E** basidia and basidioles **F** tramal hyphae **G** cheilocystidia at dissepiment edge **H** hyphae of pileipellis. Scale bars: 5 mm (**A, B**); 10 µm (**C–H**).

##### Additional specimens examined.

China, Guangdong Province, Guangzhou, Baiyun District, Maofengshan Forest Park, dead bamboo, 15 Apr 2023, Dai 24703 (BJFC042257, paratype), Dai 24704 (BJFC042258, paratype).

##### Distribution and ecology.

*Favolaschiaimbricata* is distributed in subtropical area of Guangdong Province, China; it grows on dead bamboo and causes a white rot.

#### ﻿ Favolaschia miscanthi

Taxon classificationFungiAgaricalesMycenaceae

Q.Y. Zhang, L.S. Bian, F. Wu & Y.C. Dai sp. nov.

815A8D3D-AF95-51B1-BE42-FB7F91466536

851808

Figs 3B , 5

##### Type.

China, Guangxi Autonomous Region, Hezhou, Dazhongshan Forest Park, rotten *Miscanthus*, 19 Apr 2023, Dai 24652 (BJFC042179, holotype).

##### Etymology.

“*miscanthi*” (Lat.): refers to the species inhabiting rotten *Miscanthus*.

##### Macrostructures.

Basidiomata annual, gregarious, gelatinous when fresh and dry. Pilei 3–6 × 1.5–4 mm, conchoid or semicircular; pileal surface satin white (1A1–2A1) when fresh and drying, convex, transparent with a reticulate pattern matching the pores below, glabrous; margin straight, crenulate; context thin, transparent. Hymenophore concolorous with pileal surface, poroid, up to 40 pores per basidiocarp; mature pores 0.5–1 mm diam., polygonal, larger near the base and smaller near the edge, the marginal pores often incomplete; tubes up to 0.4 mm long. Stipe absent.

##### Microstructures.

Basidiospores 7.5–10 × 5.5–7(–7.5) µm, L = 8.90 μm, W = 6.28 μm, Q = 1.37–1.46 (*n* = 60/2), broadly ellipsoid to subglobose, hyaline, thin-walled, smooth, with some guttules, faintly IKI+, CB–. Basidia 32–45 × 7–10 μm, narrowly clavate, tapered towards the base, apex broadly rounded, 4–spored, sterigmata 2–8 μm long; basidioles similar in shape to the basidia, but slightly smaller. Gloeocystidia and acanthocystidia absent. Cheilocystidia 17–34 × 7–10 μm, present at dissepiment edge, broom-shaped or irregular with small diverticulate projections on the sides and at the apex, thin-walled. Pileipellis hyphae interwoven, smooth to diverticulate, thin-walled, 4–7 µm in diam.; terminal cells cylindrical or clavate, with obtuse diverticulate projections at the apex, orientated perpendicular to pileal surface. Tramal hyphae interwoven, widely spaced in a gelatinous matrix, some with dense contents, some collapsed, thin-walled, 2–6 μm in diam. Clamp connections present.

**Figure 5.**
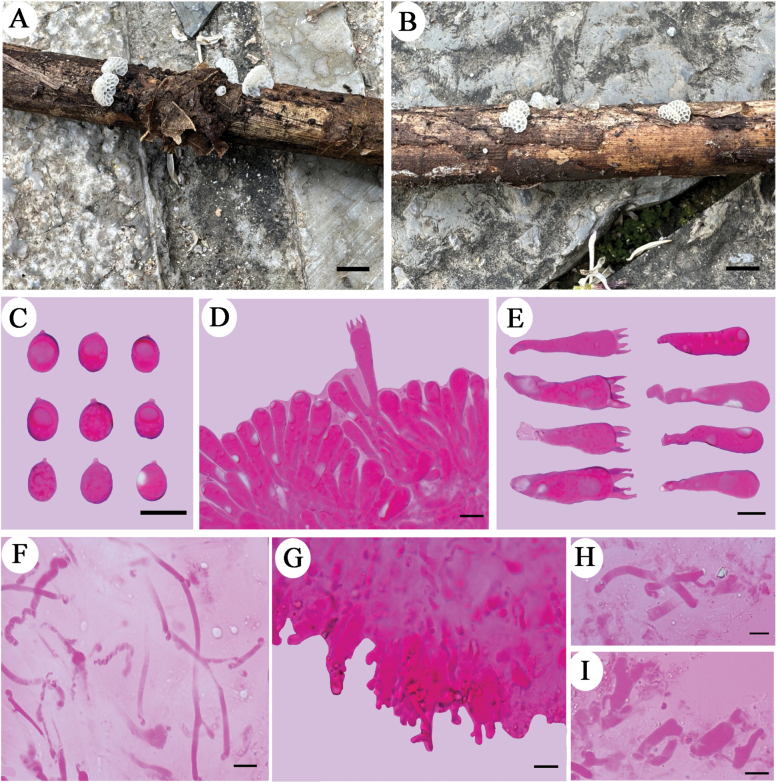
Basidiomata and microscopic structures of *Favolaschiamiscanthi***A, B** basidiomata (**A** Dai 24652, holotype **B** Dai 24653) **C** basidiospores **D** hymenium in trama **E** basidia and basidioles **F** tramal hyphae **G** cheilocystidia at dissepiment edge **H** hyphae of pileipellis **I** terminal cells of pileipellis. Scale bars: 5 mm (**A, B**); 10 µm (**C–I**).

##### Additional specimen examined.

China, Guangxi Autonomous Region, Hezhou, Dazhongshan Forest Park, rotten *Miscanthus*, 19 Apr 2023, Dai 24653 (BJFC042180, paratype).

##### Distribution and ecology.

*Favolaschiamiscanthi* is distributed in the subtropical area of the Guangxi Autonomous Region, China; it grows on rotten *Miscanthus* and causes white rot.

#### ﻿ Favolaschia sinarundinariae

Taxon classificationFungiAgaricalesMycenaceae

Q.Y. Zhang, L.S. Bian, F. Wu & Y.C. Dai sp. nov.

BE654B41-F951-5783-975B-7E53812C3958

851809

Figs 3C, D , 6

##### Type.

China, Sichuan Province, Bazhong, Nanjiang County, Micangshan National Forest Park, 25 Sep 2023, dead *Sinarundinaria*, Dai 26123 (BJFC043673, holotype).

##### Etymology.

“*sinarundinariae*” (Lat.): refers to the species inhabiting dead *Sinarundinaria*.

##### Macrostructures.

Basidiomata annual, gregarious, gelatinous when fresh and dry. Pilei 4–7 × 2–5 mm, conchoid to elliptic; pileal surface greyish-white (1B1) when fresh, dark grey (1F1) near the base upon drying, convex, transparent with a reticulate pattern matching the pores below, faintly pruinose when dry; margin straight, crenulate; context thin, opaque. Hymenophore concolorous with pileal surface, poroid, up to 80 pores per basidiomata; mature pores 0.4–1 mm diam., polygonal, the size varies irregularly, with some larger near the base or larger near the edges, the marginal pores often incomplete; tubes up to 0.5 mm long. Stipe absent.

##### Microstructures.

Basidiospores 7–9 × 5–7(–7.5) µm, L = 7.87 μm, W = 6.08 μm, Q = 1.27–1.31 (*n* = 60/2), broadly ellipsoid to subglobose, hyaline, thin-walled, smooth, with some guttules, faintly IKI+, CB–. Basidia 40–50 × 7–9 μm, narrowly clavate, tapered towards the base, apex broadly rounded, 2 or 4–spored, sterigmata 5–10 μm long; basidioles similar in shape to the basidia, but slightly smaller. Gloeocystidia and acanthocystidia absent. Cheilocystidia 15–32 × 7–10 μm, present at dissepiment edge, antler-shaped or irregular with small diverticulate projections on the sides and at the apex, thin-walled. Pileipellis hyphae interwoven, with frequent diverticulate, thin-walled, 2–4 µm in diam., swollen to 8 µm on terminal; terminal cells irregular, with obtuse diverticulate projections at the apex, orientated perpendicular to pileal surface. Tramal hyphae interwoven, widely spaced in a gelatinous matrix, some with dense contents, some collapsed, thin-walled, 1.5–3 μm in diam. Clamp connections present.

**Figure 6.**
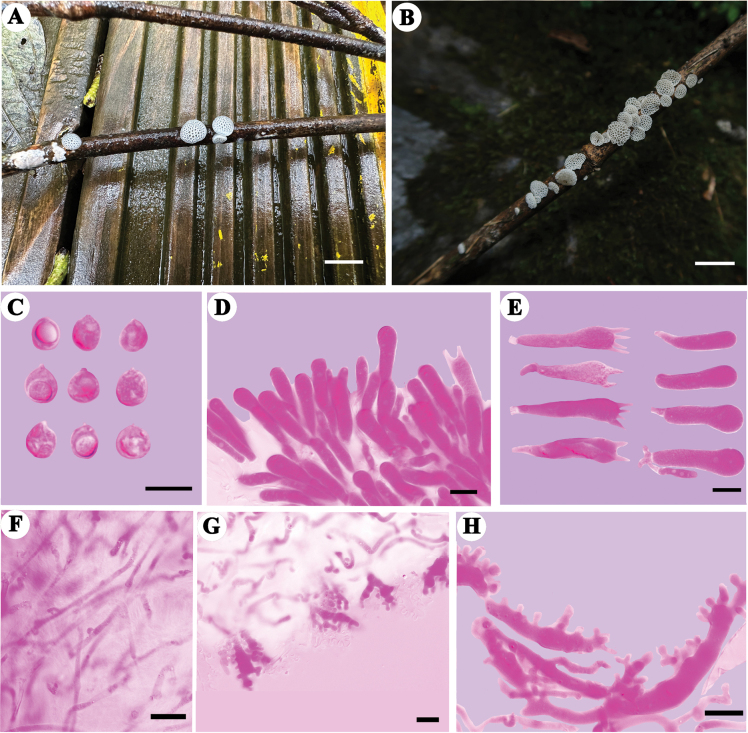
Basidiomata and microscopic structures of *Favolaschiasinarundinariae***A, B** basidiomata (**A** Dai 26123, holotype **B** Dai 26116) **C** basidiospores **D** hymenium in trama **E** basidia and basidioles **F** tramal hyphae **G** cheilocystidia at dissepiment edge **H** hyphae and terminal cells of pileipellis. Scale bars: 1 cm (**A, B**); 10 µm (**C–H**).

##### Additional specimens examined.

China, Sichuan Province, Bazhong, Nanjiang County, Micangshan National Forest Park, 25 Sep 2023, dead *Sinarundinaria*, Dai 26129 (BJFC043679, paratype), Dai 26130 (BJFC, paratype), Dai 26134 (BJFC, paratype), Dai 26136 (BJFC, paratype), Dai 26142 (BJFC043692, paratype); Leshan, Ebian County, Heizhugou, 11 Aug 2023, dead *Sinarundinaria*, Dai 26115 (BJFC043665, paratype), Dai 26116 (BJFC043666, paratype).

##### Distribution and ecology.

*Favolaschiasinarundinariae* is distributed in the subtropical area of Sichuan Province, China; it grows on rotten *Sinarundinaria* and causes white rot.

## ﻿Discussion

The earliest records of *Favolaschia* species in China date back to the 20^th^ century (Liu 1994; Liu and Yang 1994). Recently, with the application of molecular systematics, the species of *Favolaschia* in China have been continually updated (Liu 2020; Tolgor et al. 2021; Zhang and Dai 2021; Zhang et al. 2023). Morphological examination and phylogenetic analyses identified 16 species of *Favolaschia* in China (Zhang and Dai 2021; Zhang et al. 2023). In this study, three new species of *Favolaschia* are identified in south China: *Favolaschiaimbricata*, *F.miscanthi* and *F.sinarundinariae*.

Phylogenetic studies of Favolaschia largely support the section classification, based on morphology proposed by Singer (1974). Morphologically, the F.sect.Anechinus includes two subsections, F.subsect.Rubrinae and F.subsect.Depauperatae, the former lacks gloeocystidia and gloeoporous hyphae (with granular contents); the latter has these structures, while the two subsections are not well distinguished in the phylogenetic tree. The three new species, *Favolaschiaimbricata*, *F.miscanthi* and *F.sinarundinariae*, clustered within section Anechinus (group B), which is characterised by the lack of acanthocysts, gloeocystidia and gloeoporous hyphae replaced by irregular cystidia at the dissepiment edge or pileipellis.

In a combined ITS1-5.8S-ITS2-nLSU dataset-based phylogeny (Fig. 2), *Favolaschiaimbricata* grouped with another new species *F.miscanthi*, with strong support (100/1.00). However, *Favolaschiamiscanthi* can be easily distinguished from *F.imbricata* by its larger pores (0.5–1 mm vs. 0.2–0.6 mm), white pileal surface upon drying and growing on rotten *Miscanthus*, while *F.imbricata* shares dark grey or mouse-grey pileal surface upon drying and grows on dead bamboo. Based on the sub-generic classification of *Favolaschia* by Singer (1974), *F.imbricata* belongs to Favolaschiasect.Anechinussubsect.Rubrinae, because it lacks acanthocystidia, gloeocystidia and gloeoplerous hyphae.

Morphologically, *Favolaschiaimbricata* with *F.flabelliformis* and *F.tonkinensis* share grey to greyish-white basidiomata when fresh and occur in south China. However, *F.flabelliformis* differs from *F.imbricata* by its flabelliform pilei and smaller pores (0.2–0.25 mm vs. 0.2–0.6 mm, Zhang et al. (2023)). *F.tonkinensis* differs from *F.imbricata* by its larger basidiomata (6–24 mm vs. 2–5 mm) and larger basidiospores (8–12.5 × 7–10.5 µm vs. 7–9 × 5–6.8 µm, Singer (1974)). In addition, *F.imbricata* has numerous cheilocystidia (cylindrical or clavate, with obtuse diverticulate projections at the apex) at the dissepiment edge, while they are absent in *F.flabelliformis* and *F.tonkinensis*.

Morphologically, *Favolaschiamiscanthi* is characterised by its white basidiomata, inhabiting rotten *Miscanthus*, large pores (0.5–1 mm in diam.), broom-shaped cheilocystidia and broadly ellipsoid to subglobose basidiospores (7.5–10 × 5.5–7 µm) and belongs to F.sect.Anechinussubsect.Rubrinae. Four species in F.subsect.Rubrinae, viz. *F.bannaensis*, *F.rigida*, *F.semicircularis* and *F.tenuissima*, resemble *F.miscanthi* by sharing smaller (< 2 cm) and pure white to white basidiomata when fresh and occur in south China. However, *Favolaschiabannaensis* differs from *F.miscanthi* by its larger pores (up to 1.5 mm vs. up to 1 mm) and inhabit bamboo or rotten angiosperms (Zhang et al. 2023). *Favolaschiarigida* differs from *F.miscanthi* by its obvious stipe, smaller pores (0.25–0.33 mm vs. up to 0.5–1 mm) and the absence of cheilocystidia (Zhang et al. 2023). *Favolaschiasemicircularis* is readily distinguished from *F.miscanthi* by its obvious stipe and shorter basidia (24–32 μm vs. 32–45 μm in length, Zhang et al. (2023)). *Favolaschiatenuissima* differs from *F.miscanthi* by its fewer pores (up to 20 vs. up to 40) and the presence of obvious stipe (Zhang et al. 2023).

Eight specimens from Heizhugou and Micangshan National Forest Park in Sichuan Province formed a support lineage (71/1.00) and there are two base pairs differences between them in the ITS regions and no differences in morphology. We described these as a new species, namely *Favolaschiasinarundinariae*. Phylogenetically, *Favolaschiasinarundinariae* and *F.tephroleuca* cluster together in a supported clade (57/1.00) within Favolaschiasect.Anechinus, with each forming highly-supported branches. However, *F.tephroleuca* is readily distinguished from *F.sinarundinariae* by its grey to dark grey pileal surface, when fresh and fewer pores (up to 40 vs. up to 80). Morphologically, *Favolaschiaflabelliformis* is similar to *F.sinarundinariae* by sharing a similar pileal surface, the absence of stipe and almost the same size basidiospores (Zhang et al. 2023), but the former species has smaller basidiomata (2–3 mm vs. 4–7 mm), smaller pores (0.2–0.25 mm vs. 0.5–1 mm) and fewer pores (10–40 vs. up to 80, Zhang et al. (2023)). In addition, *F.flabelliformis* and *F.sinarundinariae* are phylogenetically distantly related.

During the investigations of *Favolaschia*, the information on distribution areas is obtained (Fig. 1). Regarding geographical distribution, *Favolaschia* is distributed in south China (the south Qinling-Huai River Line). In addition, an updated key to 19 species of *Favolaschia* is provided in the following.

### ﻿A key to 19 species of *Favolaschia* found in China

**Table:** 

1	Acanthocystidia present	**2**
–	Acanthocystidia absent	**5**
2	Basidiomata apricot orange when fresh	**3**
–	Basidiomata lemon chrome when fresh	**4**
3	Pilei < 5 mm	***F.minutissima***
–	Pilei > 5 mm	***F.brevibasidiata***
4	Stipe < 5 mm in length	***F.brevistipitata***
–	Stipe > 5 mm in length	***F.longistipitata***
5	Basidiomata campanulate	***F.manipularis***
–	Basidiomata conchoid, flabellate, reniform, semicircular or subcircular	**6**
6	Mature pilei > 2 cm in length	**7**
–	Mature pilei < 2 cm in length	**9**
7	Stipe present	***F.tonkinensis***
–	Stipe absent	**8**
8	Mature pores polygonal, 3–6 mm in width	***F.pustulosa***
–	Mature pores round, 1–2.3 mm in width	***F.subpustulosa***
9	Gloeocystidia present	**10**
–	Gloeocystidia absent	**11**
10	Basidiospores 4.5–5.5 μm in width	***F.peziziformis***
–	Basidiospores 7–10 μm in width	***F.crassipora***
11	Pilei transparent	**12**
–	Pilei opaque	**14**
12	Stipe present, 1–3 × 0.5–1 mm	***F.tenuissima***
–	Stipe absent	**13**
13	Basidiomata inhabiting rotten *Miscanthus*	***F.miscanthi***
–	Basidiomata inhabiting dead bamboo (Bambusoideae)	***F.bannaensis***
14	Pilei ash grey to fuscous or dark when fresh	***F.tephroleuca***
–	Pilei usually white to pale white when fresh	**15**
15	Cheilocystidia present at dissepiment edge	**16**
–	Cheilocystidia absent	**17**
16	Stipe usually present	***F.semicircularis***
–	Stipe absent	**17**
17	Mature pores 0.2–0.6 mm in width	***F.imbricata***
–	Mature pores up to 1 mm in width	***F.sinarundinariae***
18	Stipe present, with diverticulate hyphae in pileipellis	***F.rigida***
–	Stipe absent, without diverticulate hyphae in pileipellis	***F.flabelliformis***

## Supplementary Material

XML Treatment for Favolaschia imbricata

XML Treatment for Favolaschia miscanthi

XML Treatment for Favolaschia sinarundinariae

## ﻿Additional information

### Author ORCIDs

Qiu-Yue Zhang https://orcid.org/0000-0001-9458-3566

Hong-Gao Liu https://orcid.org/0000-0002-9508-3245

Wan-Ying Li https://orcid.org/0009-0006-7782-6670

Xin Zhang http://orcid.org/0009-0005-8363-7852

Yu-Cheng Dai http://orcid.org/0000-0002-6523-0320

Fang Wu https://orcid.org/0000-0002-6239-3634

Lu-Sen Bian https://orcid.org/0000-0003-3660-9056

### Data availability

The sequences are deposited in the GenBank database (Table 1).

## Supplementary materials

Supplementary material 1 Three new species of *Favolaschia* (Mycenaceae, Agaricales) from South China Data type nex Explanation note In this study, the final alignments and the retrieved topologies were deposited in TreeBASE (http://treebase.org/treebase-web/home.html), under accession ID: 30973. At the same time, we upload it as an attachment to the editor. https://binary.pensoft.net/file/1025725This dataset is made available under the Open Database License (http://opendatacommons.org/licenses/odbl/1.0/). The Open Database License (ODbL) is a license agreement intended to allow users to freely share, modify, and use this Dataset while maintaining this same freedom for others, provided that the original source and author(s) are credited.Qiu-Yue Zhang, Hong-Gao Liu, Wan-Ying Li, Xin Zhang, Yu-Cheng Dai, Fang Wu, Lu-Sen Bian
